# Mitochondrial-related microRNAs and their roles in cellular senescence

**DOI:** 10.3389/fphys.2023.1279548

**Published:** 2024-01-05

**Authors:** Ling Luo, Xingna An, Yinghui Xiao, Xiguang Sun, Sijie Li, Yingzhao Wang, Weixia Sun, Dehai Yu

**Affiliations:** ^1^ Public Research Platform, The First Hospital of Jilin University, Changchun, Jilin, China; ^2^ Department of Hand Surgery, The First Hospital of Jilin University, Changchun, Jilin, China; ^3^ Department of Breast Surgery, The First Hospital of Jilin University, Changchun, Jilin, China; ^4^ Department of Neurology, Qianwei Hospital of Jilin Province, Changchun, Jilin, China; ^5^ Department of Nephrology, The First Hospital of Jilin University, Changchun, Jilin, China

**Keywords:** cellular senescence, mitochondria, epigenetics, noncoding RNA, mitomiRs

## Abstract

Aging is a natural aspect of mammalian life. Although cellular mortality is inevitable, various diseases can hasten the aging process, resulting in abnormal or premature senescence. As cells age, they experience distinctive morphological and biochemical shifts, compromising their functions. Research has illuminated that cellular senescence coincides with significant alterations in the microRNA (miRNA) expression profile. Notably, a subset of aging-associated miRNAs, originally encoded by nuclear DNA, relocate to mitochondria, manifesting a mitochondria-specific presence. Additionally, mitochondria themselves house miRNAs encoded by mitochondrial DNA (mtDNA). These mitochondria-residing miRNAs, collectively referred to as mitochondrial miRNAs (mitomiRs), have been shown to influence mtDNA transcription and protein synthesis, thereby impacting mitochondrial functionality and cellular behavior. Recent studies suggest that mitomiRs serve as critical sensors for cellular senescence, exerting control over mitochondrial homeostasis and influencing metabolic reprogramming, redox equilibrium, apoptosis, mitophagy, and calcium homeostasis-all processes intimately connected to senescence. This review synthesizes current findings on mitomiRs, their mitochondrial targets, and functions, while also exploring their involvement in cellular aging. Our goal is to shed light on the potential molecular mechanisms by which mitomiRs contribute to the aging process.

## Introduction

Tissue and organ aging significantly contributes to a myriad of diseases, including neurodegenerative disorders, diabetes, and cancer (de Magalhães, 2013). Cellular senescence underpins this aging process, hence unlocking the mysteries of cellular senescence may hold the key to thwarting degenerative diseases.

Telomere attrition is acknowledged as an intrinsic trigger of cellular senescence, where the inevitable shortening of telomeres due to each round of cell division precipitates chromosomal instability and consequent cell cycle arrest (Hewitt et al., 2012; Williams et al., 2017; Liu, 2022). Hyperglycemic conditions have been implicated in hastening senescence in proximal tubular cells by instigating telomere reduction and activating the p53-p21-Rb signaling axis (Verzola et al., 2008; Cao et al., 2018). Similarly, oxidative stress, inducing persistent DNA damage and reactive oxygen species (ROS) formation, serves as another catalyst for cellular senescence (Kornienko et al., 2019). Hyperoxia exposure, for example, has been shown to induce DNA damage and activate senescence pathways in rat nucleus pulposus cells (Feng et al., 2017). Metabolic aberrations, such as insulin resistance and impaired glucose transport, are also associated with senescence. Age-related declines in glucose uptake and transporter expression in neuronal cells underscore this link (Jiang et al., 2013; Wang et al., 2022). Moreover, epigenetic modifications, encompassing DNA and histone alterations and noncoding RNA (ncRNA) profile changes, are central to aging and age-related pathologies (Hou et al., 2014; Fan et al., 2020; Killaars et al., 2020). Inhibition of DNA methyltransferases (DNMTs), for instance, has been found to induce senescence in multipotent stem cells and elevate aging markers (So et al., 2011).

Notably, ncRNAs, particularly microRNAs (miRNAs), are emerging as significant regulators of cellular senescence (Kim et al., 2021; Lee and Bae, 2022). These small, single-stranded ncRNAs orchestrate gene expression post-transcriptionally (Tétreault and De Guire, 2013; Duarte et al., 2015). mitomiRs, whether encoded by nuclear or mitochondrial DNA (mtDNA), are distinguished by their mitochondrial regulation, influencing metabolism and redox reactions (Guerra-Assunção and Enright, 2012; Bandiera et al., 2013; Bianchessi et al., 2015; Fan et al., 2019). Research into the mitomiR-senescence relationship is at the vanguard of cell fate determination studies. MitomiRs profoundly impact cellular senescence (Giuliani et al., 2017; Giuliani et al., 2018). Akin to their nuclear and cytoplasmic counterparts, they regulate protein expression by targeting the 3′ untranslated regions (3′-UTR) of mitochondrial mRNA (Das et al., 2012). Certain mitomiRs have been shown to exert either prooxidant or antioxidant effects in cells, regulate 16S rRNA processing, and affect bioenergetic status, with implications for tumorigenesis and progression (Bai et al., 2011; Aschrafi et al., 2012; Das et al., 2012; Sripada et al., 2017). This review aggregates and discusses findings from the PubMed database (https://pubmed.ncbi.nlm.nih.gov), probing the mechanisms by which mitomiRs mediate cellular senescence through their regulatory roles in mitochondrial function.

## Cellular senescence and tissue aging

Cellular senescence is a cornerstone of tissue aging, implicated in the deterioration of vital tissue structures crucial for normal function (Campisi, 2013). Furthermore, conditions of premature aging are marked by an increase in senescent cells, inflammatory cells, and trans-differentiated cells (Chen et al., 2015; Baker et al., 2016). Understanding the internal factors that influence cellular senescence and the consequent degeneration of tissues and organs is of paramount importance. Senescent cells enter an irreversible state of growth cessation. They typically exhibit a change in size, adopting a smoother and larger morphology compared to proliferating cells, and form senescence-associated heterochromatin foci (Narita et al., 2003). Despite their arrested growth, these cells remain metabolically active and are capable of secreting a wide array of bioactive substances such as growth factors, cytokines, chemokines, and proteases. This phenomenon is referred to as the senescence-associated secretory phenotype (SASP) (Shay and Roninson, 2004; Acosta et al., 2013; Calcinotto et al., 2019). Another hallmark of cellular senescence is the reduced expression of proteins that are critical to the electron transport chain (ETC), a change whose role as either a cause or a consequence of aging is currently debated (Gerasymchuk et al., 2020).

Prevailing research suggests that mechanisms like telomere shortening, activation of tumor suppressor pathways, oxidative stress, and mitochondrial dysfunction are key initiators of cellular senescence (Williams et al., 2017). Senescence is heralded by the attrition of telomere sequences and damage to the T-loop, triggering the DNA damage response (DDR) to double-strand breaks (Gavia-García et al., 2021). A wealth of studies support the notion that both telomere impairment and the subsequent activation of DDR signaling pathways can hasten cellular aging (White et al., 2015; Koh et al., 2020; Assis et al., 2022). In light of recent findings, the relationship between mitochondrial function and cellular senescence is garnering significant attention (Harman, 1956; Gao et al., 2022). Mitochondria are not only the cell’s energy generators but also act as critical centers for both anabolic and catabolic metabolic processes. For cells to maintain their normal functions, a stable and continuous mitochondrial response is essential, demanding precisely orchestrated biogenesis and the meticulous expression of RNAs and proteins sourced from both nuclear and mitochondrial genomes. Errors in the transport, assembly, or targeting of these mitochondrial components can lead to detrimental effects on cellular homeostasis (Wasilewski et al., 2017).

## Function of mitochondria

Mitochondria, the powerhouse organelles of the cell, are encapsulated by two distinct membranes (Duarte et al., 2014). The matrix within each mitochondrion is a hub brimming with ions, enzymes, metabolites, and crucial nucleic acids, containing its own DNA (approximately 16 kb) and RNA molecules (Choudhury and Singh, 2017). These organelles are instrumental in orchestrating a multitude of cellular functions, including cell death, autophagy, metabolic pathways, and the aging process (Weber and Reichert, 2010; Pan et al., 2011). Among their various roles, the regulation of energy metabolism is the most extensively researched. The process of oxidative phosphorylation (OXPHOS) is central to the mitochondria’s role as an energy converter, involving the synthesis of adenosine triphosphate (ATP)-the cell’s energy currency-through a sequential transfer of electrons via large protein complexes anchored in the inner mitochondrial membrane. During this intricate process, oxygen is consumed, setting up an electrochemical gradient that ultimately powers ATP synthesis. This electron transfer is facilitated by a cascade of redox-active complexes, known as complexes I through IV, which culminate in the reduction of oxygen to water (van der Bliek et al., 2017). An additional, critical function of mitochondrial respiration is the generation of ROS, which, while playing vital signaling roles at physiological levels, can become harmful in excess (Thannickal and Fanburg, 2000; Schumacker et al., 2014). These ROS can act as signaling molecules, inducing the expression and release of pro-inflammatory cytokines. Notably, this interplay between inflammatory mediators and ROS can reciprocally influence mitochondrial architecture and function, potentially establishing a detrimental feedback loop that contributes to disease pathogenesis and accelerates the aging process (Thannickal and Fanburg, 2000; Wiegman et al., 2015; Prakash et al., 2017).

## Biogenesis of miRNA

**FIGURE 1:**
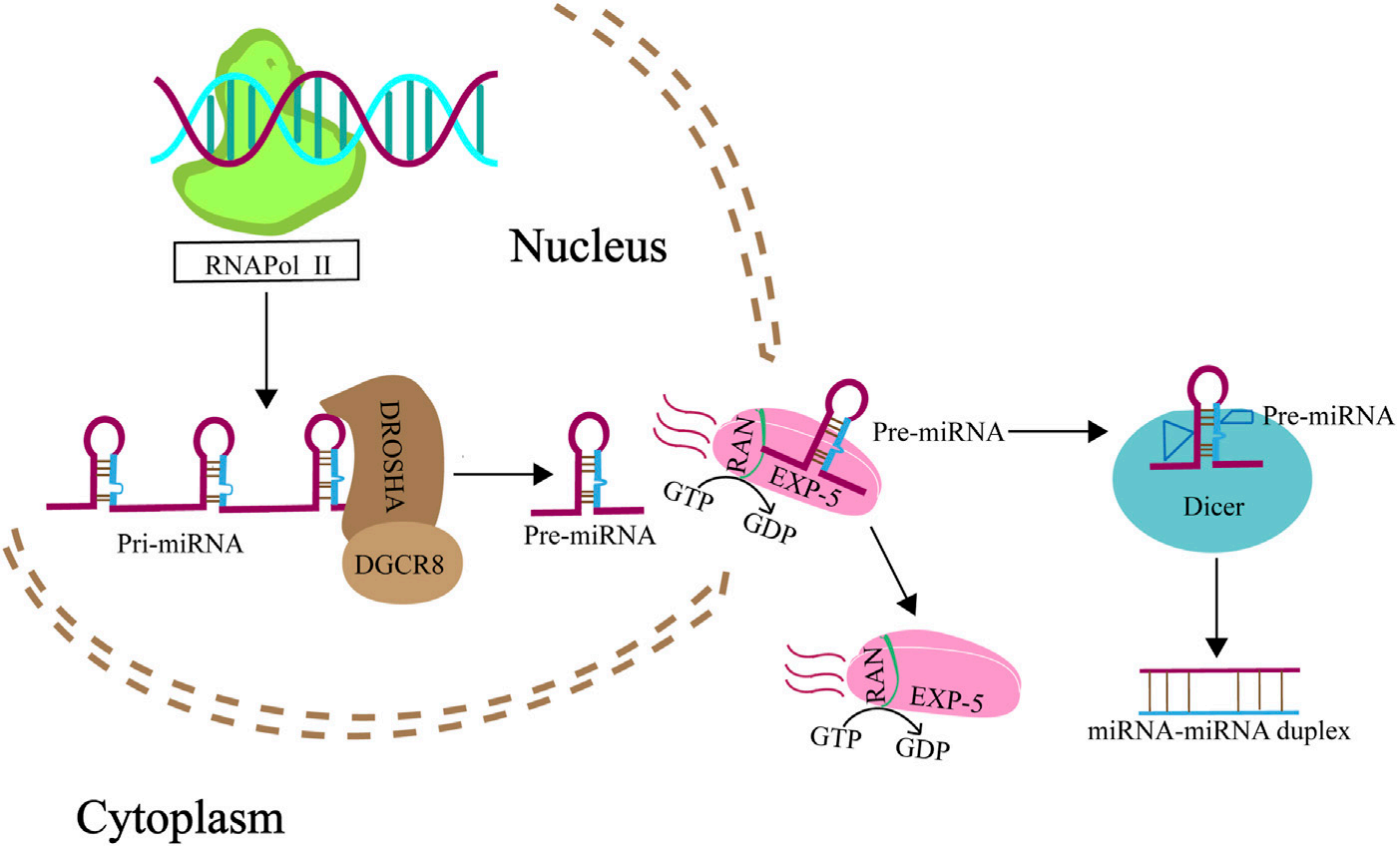
Biogenesis of miRNA. Genes that give rise to miRNAs are transcribed by RNA polymerase II from the noncoding RNA regions of the genome, yielding pri-miRNAs. These pri-miRNAs are then intricately processed into pre-miRNAs through the concerted action of the nuclear RNaseIII Drosha, and its partnering RNA-binding protein, DGCR8. Subsequently, pre-miRNAs are shuttled from the nucleus to the cytoplasm by exportin 5 in association with RanGTP (A GTP-binding nuclear protein), passing through the nuclear pore complexes. Once in the cytoplasm, these pre-miRNAs encounter Dicer, another RNase III enzyme, which meticulously cleaves them into mature miRNA duplexes.

miRNAs are single-stranded, noncoding RNAs ranging from 19 to 24 nucleotides in length, acting as pivotal regulators of gene expression at the post-transcriptional level. They modulate gene expression by binding to complementary sequences within the 3′ untranslated regions (3′UTR) of target mRNAs, leading to mRNA degradation or inhibition of translation (Bartel, 2004). The biosynthesis of miRNAs is a complex process involving several enzymatic steps within both the nucleus and the cytoplasm (Figure 1) (Ha and Kim, 2014; Lin and Gregory, 2015). Typically, miRNA (non-mitomiR) biogeesis begins in the nucleus with the transcription of a primary-miRNA (pri-miRNA) from the DNA. This pri-miRNA is then processed by the Microprocessor complex, which consists of ribonuclease III enzyme (RNaseIII) and the DiGeorge syndrome chromosomal region 8 (DROSHA/DGCR8) enzyme, resulting in a precursor miRNA (pre-miRNA). This pre-miRNA is subsequently transported to the cytoplasm by the nuclear export factor exportin 5 in complex with the GTP-binding nuclear protein RAN-GTP. Once in the cytoplasm, the pre-miRNA is further cleaved by another RNase III enzyme, Dicer, to produce a double-stranded, mature miRNA (Srinivasan and Das, 2015).

## Transportation of miRNA into mitochondria

**FIGURE 2:**
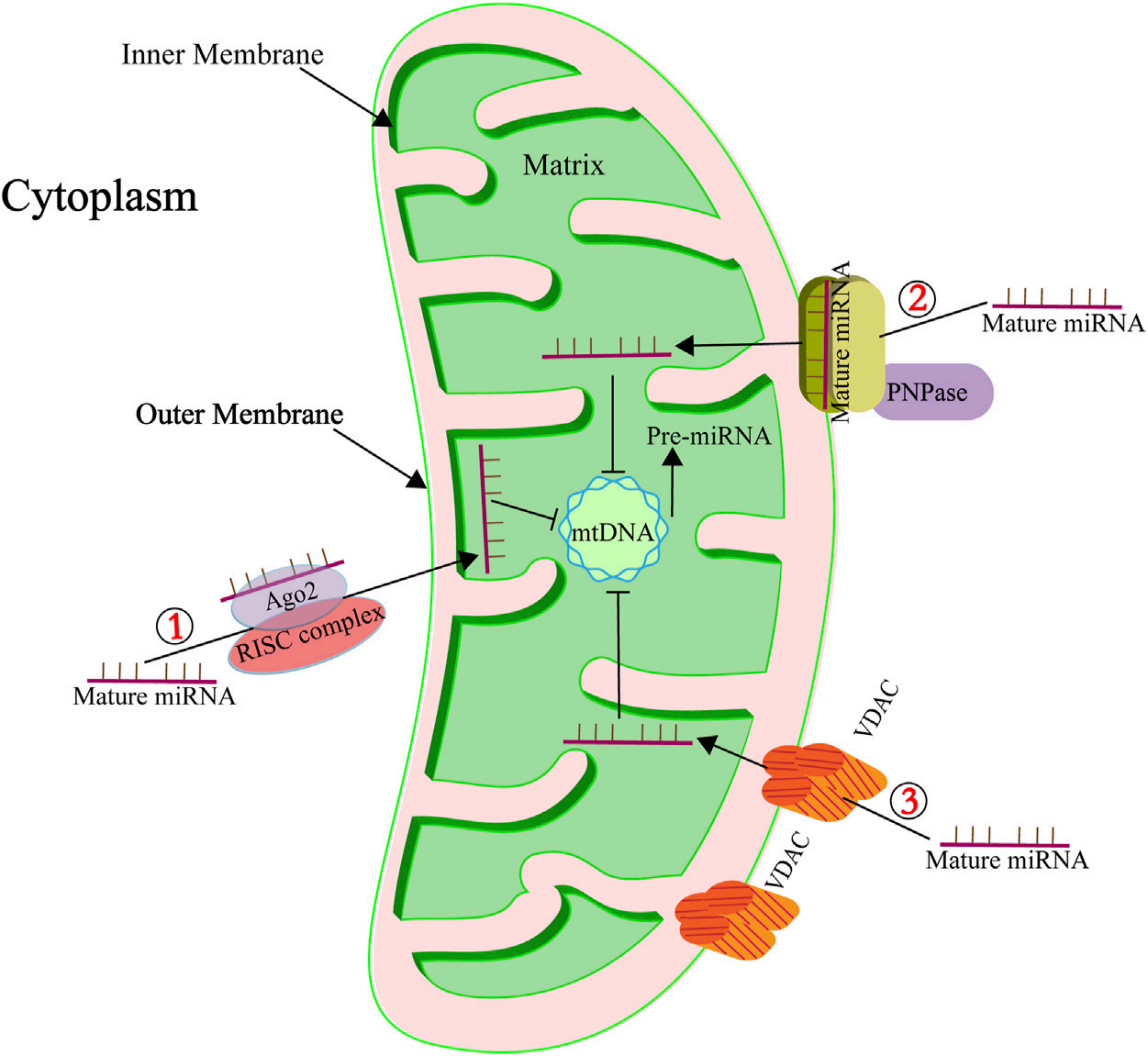
Key mediators of miRNA mitochondrial import. ① Highlights the role of Ago2 in the subcellular distribution and transport of mitomiRs. Ago2, known for its RNase activity, functions as a crucial component of the RISC. ② Denotes the involvement of PNPase in the transportation of mitomiRs. PNPase is adept at recognizing specific structural features of housekeeping ncRNAs, facilitating their correct folding, passage through the mitochondrial membrane, and eventual reacquisition of their native conformation within the mitochondrial matrix. ③ Points to the role of the pore-forming protein VDAC in ncRNA transport. To date, VDAC’s role in RNA import is supported by a singular study indicating its involvement in the translocation of tRNA into mitochondria (Salinas et al., 2006).

miRNAs have been detected within mitochondria (Lung et al., 2006; Chan et al., 2009; Kren et al., 2009; Bian et al., 2010; Favaro et al., 2010; Barrey et al., 2011; Huang et al., 2011; Shinde and Bhadra, 2015; Fan et al., 2019), and a particular subset, termed mitomiRs, are known to finely regulate mitochondrial functions (Li et al., 2012). These mitomiRs, predominantly encoded by the nuclear genome, are imported into mitochondria to modulate the expression of mRNAs originating from the mitochondrial genome. Table 1 provides a comprehensive list of these nuclear-derived mitomiRs and their mitochondrial roles. The precise mechanisms facilitating miRNA import into mitochondria remain elusive, but several proteins have been implicated in this complex transport process, as depicted in Figure 2. One of the key proteins is Argonaute2 (Ago2), which is ubiquitously present in mitochondria across various cell types. Research indicates a pivotal role for miRNA-Ago2 interactions in the mitochondrial translocation of mitomiRs (Fasanaro et al., 2008; Latronico and Condorelli, 2012; Meloni et al., 2013; Wang et al., 2015). For instance, Zhang et al. (2014) demonstrated that miR-1, which is upregulated during myogenesis, can effectively penetrate mitochondria by forming a complex with Ago2, thereby enhancing the translation of mitochondrial-encoded transcripts. Gohel et al. (2021) investigated the role of mitochondrial miRNAs in the pathogenesis of Fragile X-associated tremor/ataxia syndrome (FXTAS). In their study, miR-320a was found to associate with Ago2 in HEK293 cells exhibiting expanded CGG repeats, with a notable enrichment of this complex within the mitochondrial matrix. The miRNA-Ago2 complex associates with the RNA-induced silencing complex (RISC) and can be shuttled into mitochondria via the coordinated action of the sorting and assembly machinery (SAM50), translocase of the outer mitochondrial membrane 20 (TOM20), and translocase of the inner mitochondrial membrane (TIM) (Jusic et al., 2020). Another significant player is polynucleotide phosphorylase (PNPase), located at the inner mitochondrial membrane and projecting into the intermembrane space. PNPase has been identified as a crucial component in miRNA mitochondrial import. Wang et al. found that disrupting the PNPase gene (*pnpt1*) perturbs mitochondrial morphology and function in murine hepatic cells, partly by impeding RNA imports that govern the transcription and translation of ETC proteins (Wang et al., 2010). Shepherd et al. (2017) further elucidated that PNPase overexpression in HL-1 cardiomyocytes correlates with increased mitochondrial miRNA-378 levels, underscoring its role in miRNA transport. The voltage-dependent anion channel (VDAC) constitutes another potential conduit for miRNA import. As a highly conserved and predominant protein in the mitochondrial outer membrane, VDAC is hypothesized to facilitate the translocation of small noncoding RNAs into mitochondria. While Salinas et al. (2006) have shown that plant mitochondrial VDAC can bind to tRNA and mediate its import *in vitro*, its involvement in miRNA transport remains to be thoroughly investigated, indicating an exciting direction for future research (Colombini, 1980; Das et al., 2008; Bandiera et al., 2013; Macgregor-Das and Das, 2018a).

**TABLE 1:** Nuclear-encoded mitomiRs and their functional roles in mitochondria.

mitomiRs	Target genes	Cell/tissue	Modulation of mitomiR	Function	Reference
miR-1	*mt-COX1*, *mt-ND1*	C_2_C_12_ cells	↑	Enhance protein synthesis and ATP production	Zhang et al. (2014)
miR-21-5p	*mt-CYTB*, *mt-ND1*	Spontaneous hypertensive rats, human muscular cells	↑ or ↓	Enhance Cytb translation in mitochondria	Barrey et al. (2011), Li et al. (2016)
miR-146a-5p	*mt-ND1*, *mt-ND2*, *mt-ND4*, *mt-ND5*, *mt-ND6*, *mt-ATP8*	206ρ cells	↑	—	Dasgupta et al. (2015), Giuliani et al. (2017)
miR-151a-5p	*mt-CYTB*	Severe asthenozoospermia	↑	Regulate ATP production through targeting Cytb	Zhou et al. (2015)
miR-181-c	*mt-COX1*, *mt-COX2*	Rat cardiomyocytes	↑	Increase ROS generation, causing ETC, complex IV remodeling	Das et al. (2012), Das et al. (2014)
miR-378	*mt-ATP6*	HL-1 cells	↑	Decrease the functionality of ATP synthase	Jagannathan et al. (2015)
miR-762	*mt-ND2*	Mouse cardiomyocytes	↓	Improve OXPHOS efficiency (ADP/oxygen)	Yan et al. (2019)
miR-2392	*mt-ND4*, mt-*CYTB*, *mt-COX1*	TSCC cells, CAL-27 cells	↓	Downregulate OXPHOS and upregulate glycolysis	Fan et al. (2019)
miR-5787	*mt-COX3*	TSCC cells	↓	Attenuate OXPHOS and enhance glycolysis	Chen et al. (2019)
miR-92a	*mt-CYTB*	db/db mice heart	↓	—	Li et al. (2019a)
let-7b-5p, miR-34b-5p, let-7c-5p, miR-324-3p, miR-324-5p, miR-454-3p	*mt-COX1*, *mt-COX2*, mt-*ATP6*, *mt-ATP8*, *mt-ND5*, *mt-ND6*	Human primary myoblast	↓	—	Barrey et al. (2011)
miR-15a, miR-196a, miR-296-3p	*mt-ND2*, *mt-ND4*, *mt-ND4L*, *mt-ND5*, *mt- ATP6*	RAS-STCs	↑	Impair mitochondrial structure and function in swine STCs	Farahani et al. (2020)

↑, increase; ↓, decrease. ATP, adenosine-triphosphate; ADP, adenosine -diphosphate; ROS, reactive oxygen species; OXPHOS, oxidative phosphorylation; ETC, electron transport chain; RAS, renal artery stenosis; STCs, scattered tubular-like cells; TSCC, tongue squamous cell carcinoma; COX, cytochrome c oxidase subunit. CYTB, cytochrome B. ND, NADH, dehydrogenase. The italicized text represents gene names.

## mitomiRs that are transcribed from the mitochondrial genome

Emerging research has unveiled a complex landscape of noncoding RNA within mitochondria, including the discovery of novel mitomiRs that target the UTRs of mitochondrial genes (Lung et al., 2006; Villegas et al., 2007; Burzio et al., 2009; Mercer et al., 2011; Rackham et al., 2011; Smalheiser et al., 2011). Reports suggest that miR-1974, miR-1977, and miR-1978 may be transcribed directly from mtDNA (Bandiera et al., 2011; Barrey et al., 2011; Sripada et al., 2012; Kim et al., 2017). Supporting this notion, the sequences of certain pre-miRNAs and mature miRNAs-such as pre-miR-let7 and pre-miR-302a, which have been identified in human muscle mitochondria-show alignment with the mitochondrial genome, hinting at the possibility of intramitochondrial mitomiR biosynthesis (Barrey et al., 2011; Murri and El Azzouzi, 2018; Kussainova et al., 2022). However, the verification of these mitomiRs as products of mitochondrial gene transcription remains elusive. The lack of canonical miRNA processing enzymes-Drosha, DGCR8, and Dicer-within mitochondria presents a hurdle in confirming the mitochondrial origin of these miRNAs (Macgregor-Das and Das, 2018b). Table 2 compiles a list of mitomiRs that are postulated to be transcribed from the mitochondrial genome. The existence and functions of these miRNAs are primarily predicted using bioinformatics tools like TargetScan, MiRanda, and miRBase, with most awaiting experimental confirmation of their roles.

**TABLE 2:** List of mitomiRs identified through bioinformatics prediction.

mitomiR	miRNA hosting genes	Location on mtDNA	Cells	Validation status	Functional confirmation	Reference
hsa-miR-1974	*mt-ND6/mt-TRNE*, *mt-ND5*, *mt-CYTB*, *mt-ND4*, *mt-ATP6 mt-ND1*, *mt-TRNS2/mt-TRNL*	14,675–14,697	Hela cells	Validated	Unknown	Bandiera et al. (2011), Sripada et al. (2012)
hsa-miR-1977	*mt-ND4*, *mt-TRNN*, *mt-TRNP*, *mt-ND2*, *mt-RNR2*, *mt-ND5*, *mt-TRNL2*	5,693–5,714	Hela cells	Predicted	Unknown	Bandiera et al. (2011), Sripada et al. (2012)
hsa-miR-1978	*mt-ND1*, *mt-COX2*, *mt-COX1*	654–674	Hela cells	Predicted	Unknown	Bandiera et al. (2011), Sripada et al. (2012)
hsa-miR-4485	*mt-16S rRNA*	2,562–2,582	HEK293, Hela and MCF7	Validated	Unclear	Sripada et al. (2012), Sripada et al. (2017), Farfan et al. (2021)
hsa-miR-4461	*mt-ND4L*	10,690–10,712	HEK293, Hela	Predicted	Unknown	Sripada et al. (2012)
hsa-miR-4463	*mt-ND5*	13,050–13,068	HEK293, Hela	Predicted	Unknown	Sripada et al. (2012)
hsa-miR-4484	*mt-L-ORF*	5,749–5,766	HEK293, Hela	Predicted	Unknown	Sripada et al. (2012)
hsa-miR-mit-1	*mt-COX1*	6,715–6,735	Human skeletal muscle myoblast cells	Predicted	Unknown	Shinde and Bhadra (2015)
hsa-miR-mit-2	*mt-ATP8*	8,454–8,472	Human skeletal muscle myoblast cells	Predicted	Unknown	Shinde and Bhadra (2015)
hsa-miR-mit-3	*mt-ATP6*	9,186–9,207	Human skeletal muscle myoblast cells	Predicted	Unknown	Shinde and Bhadra (2015)
hsa-miR-mit-4	*mt-ND4L*	10,832–10,851	Human skeletal muscle myoblast cells	Predicted	Unknown	Shinde and Bhadra (2015)
hsa-miR-mit-5	*mt-ND2*	5,094–5,115	Human skeletal muscle myoblast cells	Predicted	Unknown	Shinde and Bhadra (2015)
hsa-miR-mit-6	*mt-16S rRNA*	2,406-2,426	Human skeletal muscle myoblast cells	Predicted	Unknown	Shinde and Bhadra (2015)

The italicized text represents gene names.

## Mitochondrial-related miRNAs and aging

### Mitochondrial dysfunction and cellular senescence

**FIGURE 3:**
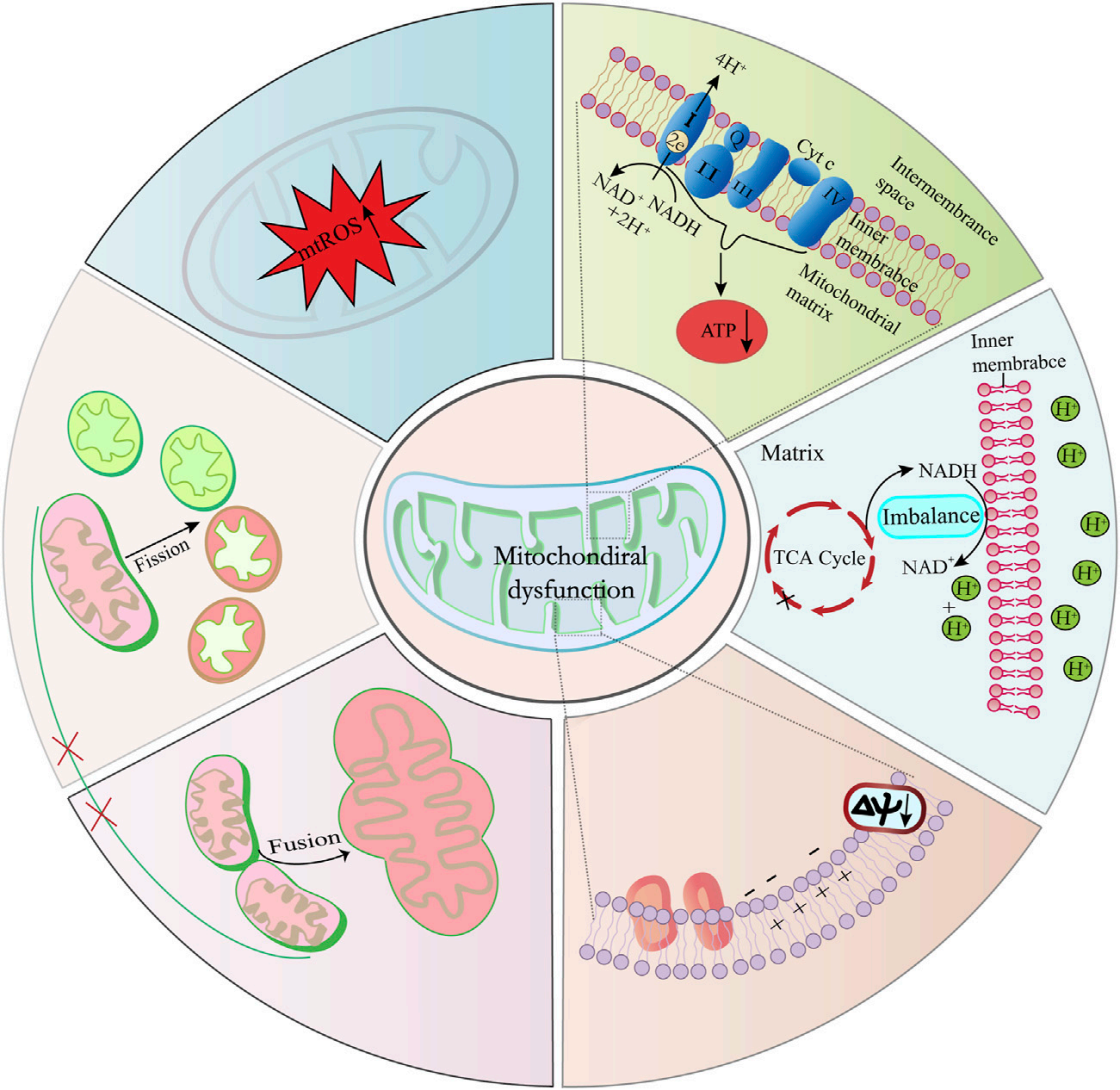
Key factors contributing to mitochondrial dysfunction and cellular senescence. Imbalances in the mitochondrial NAD+/NADH ratio, elevated ROS levels, and diminished ETC, complex activities are primary contributors to mitochondrial dysfunction. Furthermore, disturbances in the equilibrium of mitochondrial division and fusion processes result in structural and functional alterations of mitochondria.

Mitochondrial dysfunction is increasingly recognized as a catalyst for accelerated aging and a contributor to age-related diseases (Sastre et al., 2000; Trifunovic et al., 2004). This dysfunction manifests through a spectrum of features, including: 1) diminished activity of ETC complexes; 2) disrupted NAD+/NADH balance; 3) an upset in the delicate equilibrium between mitochondrial fission and fusion processes; 4) elevated mitochondrial-reactive oxygen species (mtROS) production; and 5) compromised mitochondrial membrane potential alongside changes in mitochondrial permeability (refer to Figure 3). These dysfunctions coalesce to impair mitochondrial ATP production, a deficit that becomes particularly evident in the mitochondria of aged skeletal muscle, cardiac, and adipose tissues (Peterson et al., 2012; Boengler et al., 2017). The resulting disrupted energy metabolism, particularly the altered NAD+/NADH ratio, has a profound impact, not only on mitochondrial efficiency but also on cellular health, as it has been implicated in triggering cellular senescence (Stein and Imai, 2014; Wiley et al., 2016; Bakalova et al., 2022; Miwa et al., 2022).

Maintaining a functional mitochondrial network through balanced fission and fusion is critical for mitochondrial integrity and function, thereby preventing damage accumulation and coordinating with autophagy for quality control (Ham and Raju, 2017). The importance of these processes is highlighted by findings such as those of Li X. et al. (2019), who observed that fibroblast growth factor 21 (FGF21) deficiency can induce mitochondrial fusion and senescence in human mesenchymal stem cells (hMSCs). Conversely, Rana et al. (2017) reported that enhancing dynamin-related protein 1 (Drp1)-dependent mitochondrial fission could extend the healthy lifespan in *Drosophila* Moreover, Yamamoto-Imoto et al. (2022) elucidated the interplay between cellular senescence and autophagy in renal aging, demonstrating that the transcription factor Mondo A might safeguard against senescence by promoting autophagy and preserving mitochondrial homeostasis.

Mitochondrial dysfunction is implicated in various metabolic disorders, and it’s particularly noted for its role in the aging process. One critical way mitochondria contribute to aging is through the heightened production of ROS. Normally, ROS generation is balanced with its neutralization within the body. However, when this balance is disturbed, and ROS production becomes excessive, it can overwhelm cellular antioxidant defenses. This imbalance leads to oxidative stress, which can precipitate premature aging and damage to tissues and organs (Chiang et al., 2020; Marí et al., 2020; Sreedhar et al., 2020; Zhou et al., 2021). Intriguing research by Zhu et al. (2022) has revealed that interleukin-13 (IL-13) treatment may prompt cellular aging in submandibular gland-C6 cells. They propose a mechanism where IL-13 elevates levels of phosphorylated signal transducer and activator of transcription 6 (p-STAT6) and mtROS. Concurrently, it diminishes the mitochondrial membrane potential and the production of ATP, alongside reducing both the expression and activity of superoxide dismutase 2 (SOD2), an essential mitochondrial antioxidant enzyme (Zhu et al., 2022). Moreover, Springo et al. (2015) have shown that mtROS production is naturally increased in the smooth muscle cells of aged mice under hypertensive conditions. Therefore, targeted repair or enhancement of the cellular antioxidant system is an effective approach to reducing mitochondrial oxidative damage and delaying cellular aging. Park et al. (2013) have observed that supplementing the diet of elderly dogs with astaxanthin leads to increased ATP production, enhanced mitochondrial mass, and heightened activity of cytochrome c oxidoreductase, along with a reduction in mtROS production in leukocytes. These effects collectively may enhance mitochondrial functionality and mitigate oxidative damage to cellular DNA and proteins (Park et al., 2013). Glutathione (GSH) is crucial in shielding cells from damage induced by oxidative stress. Dysfunctional glutathione mechanisms are associated with the onset of numerous diseases as part of the aging process. An elevation in ROS levels, a reduction in GSH reserves, disruption of intracellular oxidoreductases, or diminished thioredoxin activity can all result in an imbalance between cellular ROS and GSH, subsequently leading to cellular aging (Liu et al., 2022). Ryan et al. (2010) have reported that resveratrol elevates GSH levels and boosts the activity of manganese superoxide dismutase (MnSOD) and catalase. MnSOD, which is situated within the mitochondrial matrix, plays a pivotal role in safeguarding mitochondria against oxidative damage. Furthermore, resveratrol reduces the production of oxidants and mitigates oxidative damage in the gastrocnemius muscles of both young adult and aged mice engaged in exercise (Ryan et al., 2010).

### Mitochondrial-localized miRNAs and their impact on mitochondrial dysfunction

The presence of miRNAs within the mitochondria endows them with the ability to modulate mitochondrial functions, thereby enriching our comprehension of miRNA characteristics and their influence on cellular destiny. Research into mitomiR-mediated mitochondrial regulation is prevalent, particularly in age-associated diseases, including cancers (Giuliani et al., 2017; John et al., 2020; Singh and Storey, 2021; Erturk et al., 2022; Kuthethur et al., 2022; Rivera et al., 2023). Liao et al. (2022) elucidated that mitomiR-1285 induces mitochondrial dysfunction and mitophagy in jejunal epithelial cells exposed to copper by promoting mtROS accumulation and reducing mitochondrial membrane potential. They also established that mitomiR-1285 targets isocitrate dehydrogenase [NADP (+)] 2 (IDH2), exacerbating copper-induced mitochondrial damage by repressing IDH2 via its 3′UTR (Liao et al., 2022). Kuthethur and colleagues uncovered 13 mitomiRs encoded by the mitochondrial genome, noting variations in expression across different breast cancer cell lines. Among these, mitomiR-5-5p was markedly upregulated in all examined cell lines, with mt-COX1 and mt-COX2 identified as its targets (Kuthethur et al., 2022). In tongue squamous cell carcinoma, overexpression of mitomiR-2392 was shown to selectively inhibit mtDNA transcription, significantly reducing the expression of ND4, COX1, and CYTB, which in turn suppresses OXPHOS (Fan et al., 2019). Shu et al. (2018) noted a decrease in miR-107 levels in mice with AD-like symptoms. Meanwhile, other studies by John et al. (2020) and Rech et al. (2019) have demonstrated that decreased miR-107 levels can lead to mitochondrial dysregulation, characterized by reductions in mitochondrial membrane potential and ETC activity (Shu et al., 2018; Rech et al., 2019; John et al., 2020). Ahn et al. (2021) discovered miR-494-3p in the mitochondria of retinal pigmented epithelial cells, where it plays a role in regulating mitochondrial function. Depletion of miR-494-3p led to a decline in ATP production and mitochondrial membrane potential, with mitochondrially encoded cytochrome c oxidase subunit 3 (mt-COX3) mRNA proposed as a likely target of miR-494-3p (Rehmsmeier et al., 2004; Ahn et al., 2021). Further investigations indicated that miR-15a, miR-196a, and miR-296-3p, residing in the mitochondria of scattered tubular-like cells, partake in the post-transcriptional governance of genes integral to mitochondrial function. Predictive analyses suggest these miRNAs target mitochondrial DNA to downregulate *mt-ND2*, *mt-ND4, mt-ND4L*, *mt-ND5*, and *mt-ATP6*, impairing mitochondrial integrity and activity (Farahani et al., 2020). Collectively, these studies suggest that mitomiRs predominantly influence mitochondrial function by affecting various aspects of metabolic pathways, such as the TCA cycle and the ETC. Mechanistically, mitomiRs may suppress protein synthesis by targeting mRNAs within mitochondria via their 3′UTRs, akin to their action in the cytoplasm. To date, there is no evidence to suggest that mitomiRs operate distinctively from other miRNAs.

### Role of mitochondrial-related miRNAs in aging

Extensive research has illuminated the association between variations in mitomiRs and cellular aging. These variations in mitomiRs can lead to the accumulation of mtROS, activation of the mitochondrial apoptotic pathway, inflammation, and alterations in mitochondrial dynamics-all pivotal in driving cellular senescence.

Mitochondrial complex I dysfunctions resulting in mtROS production are recognized as a key aging hallmark (Murphy, 2009; Ugalde et al., 2010). For instance, Lang et al. (2016) discovered that miR-15b levels diminish during senescence in human dermal fibroblasts, which is induced by ultraviolet or gamma irradiation. They found that miR-15b inhibition upregulated SIRT4 expression, heightening mtROS generation, lowering mitochondrial membrane potential, and altering the expression of nuclear-encoded mitochondrial genes and the SASP components (Lang et al., 2016). Das et al. (2012) highlighted how miR-181c, encoded by nuclear DNA, migrates into mitochondria, suppressing mt-COX1 and triggering mitochondrial dysfunction in rat ventricular myocytes. This led to restructured respiratory complex IV, increased mtROS, and induced myocardial dysfunction (Das et al., 2012; Das et al., 2017). Moreover, delivering miR-181c into rat hearts impeded exercise capacity and provoked heart failure symptoms (Das et al., 2014).

Oxidative stress, a major aging contributor, damages mitochondria, generating excessive mtROS, triggering cellular damage, and prompting apoptotic pathways. Sripada et al. (2017) showcased miR-4485’s involvement in regulating mitochondrial functions, demonstrating its direct interaction with mitochondrial 16S rRNA. This interaction impacted pre-rRNA processing and protein synthesis, which in turn modulated mitochondrial complex I activity, ATP, ROS levels, and induced apoptosis in cancer cells (Sripada et al., 2017). The expression of mitomiRs like miR-181a, −34a, and −146a, has been found to increase and localize within mitochondria in senescent endothelial cells, regulating apoptosis sensitivity by modulating Bcl-2 and activating caspases (Giuliani et al., 2018).

Senescent cells contribute to the acceleration of inflammaging by secreting proinflammatory factors, which plays a critical role in fostering the onset of prevalent diseases associated with aging. Giuliani et al. (2017) dicussed the potential influence of mitomiRs on senescent cells’ energetic, oxidative, and inflammatory status, with specific attention to mitomiRs like let-7b, miR-1, and miR-146a-5p. Among these, miR-146a is particularly noteworthy for its association with inflammation-mediated aging, although not all studies have confirmed its translocation to mitochondria (Giuliani et al., 2017). Research by Wang et al. (2015) has shed light on the behavior of miR-146a in the context of a rat traumatic brain injury model, where a significant compartmental shift of miR-146a from mitochondria to the cytoplasm was observed alongside other mitochondria-enriched miRNAs. This redistribution is intricately linked to trauma-induced alterations in mitochondrial bioenergetics and the regulation of inflammatory markers. Their further studies illustrated that the targeted delivery of miR-146a via nanoparticles markedly reduces the production of inflammatory mediators both *in vitro* and *in vivo* (Wang et al., 2015; Wang WX. et al., 2017; Wang et al., 2021). Through bioinformatics analysis, Rippo et al. (2014) discussed the role of inflammation-related mitomiRs in human “inflame-aging” and predicted that miR-181a, miR-34a, and miR-146a might regulate mitochondrial function and inflammation during cellular aging by modulating Bcl-2 family members (Rippo et al., 2014). Li et al. (2010) found that the upregulation of miR-146a could curb the production of inflammatory mediators in senescent cells, thus limiting their harmful impact on surrounding tissues (Li et al., 2010). Similarly, Olivieri et al. (2013) and Ong et al. (2018) demonstrated that an increase in miR-146a is linked with inflammatory senescence in various cell types, including human fibroblasts, trabecular meshwork cells, and endothelial cells (Olivieri et al., 2013; Ong et al., 2018).

Mitochondrial dynamics are crucial for maintaining cellular integrity and play a pivotal role in regulating senescence and related cellular processes. The critical balance of mitochondrial fusion and fission is primarily controlled by essential proteins like mitofusin-1 (MFN1), MFN2, optic atrophy 1 (OPA1), and mitochondrial fission factor (MFF) (Ni et al., 2015). Numerous studies indicate that mitochondria-related miRNAs modulate these proteins, thereby influencing mitochondrial function. Mu et al. provided evidence that miR-20b negatively influences the expression of MFN1 and MFN2, the principal mediators of mitochondrial fusion (Mu et al., 2021). Bucha et al. demonstrated in a model of Huntington’s disease that miR-214 directly targets MFN2, with its upregulation leading to decreased MFN2 levels, thus disturbing the mitochondrial fusion-fission balance, resulting in impaired fusion and increased fragmentation (Bucha et al., 2015). In their work, Lang et al. (2017) showed that enhancing SIRT4 expression via miR-15b inhibition leads to elevated levels of L-OPA1, thereby fostering mitochondrial fusion (Lang et al., 2017). Conversely, Lee et al. (2017) identified that miR-200a-3p binds MFF mRNA, reducing MFF expression, which in turn promotes mitochondrial elongation and influences overall mitochondrial dynamics. Goljanek-Whysall et al. (2020) highlighted the role of miR-181a as a regulator of mitochondrial dynamics, particularly during the aging of skeletal muscle. They demonstrated that in aged mice, miR-181a administration increased the expression of mitochondrial genes such as COX1 and ND-1, while a decline in miR-181a with age correlated with an accumulation of autophagy-related proteins and the presence of dysfunctional mitochondria (Goljanek-Whysall et al., 2020).

## Conclusion and perspectives

Previous research on mitochondria has primarily focused on their role in energy provision and metabolic regulation. However, the latest findings suggest that mitochondria also serve as critical sites for cellular signal transduction and information exchange. The cross-talk and information network between the nucleus, cytoplasm, and mitochondria significantly influence vital cellular processes such as survival, apoptosis, differentiation, and aging.

Recent studies have shown that under certain conditions, such as disease or stress, noncoding RNAs, especially miRNAs, are abnormally enriched in mitochondria (Wang X. et al., 2017; Carden et al., 2017; Wang et al., 2021; Yan et al., 2021). This suggests that these mitochondria-enriched miRNAs play a crucial role in regulating mitochondrial behavior, which could significantly affect cellular functions, including drug resistance, inflammation, and aging.

miRNAs are small regulatory molecules that are abundant and diverse within cells. They are relatively easy to transcribe, synthesize, transport, and degrade. These characteristics make miRNAs well-suited for shuttling between the nucleus and mitochondria, transferring signals, and flexibly performing regulatory functions within mitochondria. The study of miRNA functions within the nucleus and cytoplasm is vast. However, research into mitomiRs has emerged as a new hot topic in recent years. Elucidating the molecular mechanisms of mitomiR regulation of mitochondrial behavior can expand our understanding of miRNA functions and more deeply clarify the connections between miRNA functions and diseases.

Nevertheless, research on mitomiRs is still in its infancy, with few reports published. Integrating current literature on mitochondrial-related miRNAs and mitomiRs, we identify several unclear aspects: 1) the molecular mechanisms and driving forces behind the translocation of mitomiRs into mitochondria; 2) the varieties and amounts of mitomiRs transcribed by mtDNA; 3) how mitomiR profiles change during biological events and their impact on mitochondrial behaviors; and 5) the significance and influence of mitochondrial-cytoplasmic-nuclear communication. These areas are gaps that future researchers will need to fill.

The study of mitomiRs is undoubtedly more challenging than that of nuclear and cytoplasmic miRNAs. This difficulty is due, in part, to the lack of efficient techniques to edit genetic material in mitochondria, which poses obstacles to the study of mitochondrial genetics and epigenetics. Although next-generation sequencing technology has become widely used, there is still a lack of experience in the bioinformatic analysis of mitomiRs. Furthermore, during our research group’s investigations into mitomiRs, we have found that isolating relatively pure mitomiRs is not an easy task. This could be another barrier to understanding the biological functions of mitomiRs.

In summary, in this article, we review the currently discovered mitochondrial-related miRNAs and their impact on mitochondria and cellular function, particularly regarding cellular aging. We hope this review will attract more researchers to focus on the investigation of mitochondrial-related miRNAs and develop their potential applications in disease diagnosis and treatment in the future.

## Figures and Tables

**Table:** 

**miRNA**	MicroRNA
**mtDNA**	Mitochondrial DNA
**mitomiR**	Mitochondrial miRNA
**ncRNA**	non-coding RNA
**3′-UTR**	3′ untranslated regions
**SASP**	Senescence-associated secretory phenotype
**DDR**	DNA damage response
**D-loop**	Displacement loop
**ATP**	Adenosine triphosphate
**ROS**	Reactive oxygen species
**DROSHA/DGCR8**	DiGeorge syndrome chromosomal region 8
**RANGTP**	A GTP-binding nuclear protein
**RNaseIII**	Ribonuclease III enzyme
**Ago2**	Argonaute2
**FXTAS**	Fragile X-associated tremor/ataxia syndrome
**RISC**	RNA-induced silencing complex
**SAM50**	Sorting and assembly complex
**TOM20**	Translocase of the outer mitochondrial membrane 20
**TIM**	Translocase of the inner mitochondrial membrane
**PNPase**	Polynucleotide phosphorylase
**VDAC**	Voltage-dependent anion channel
**ADP**	Adenosine-diphosphate
**CYTB**	Cytochrome B
**ND**	NADH dehydrogenase
**RAS**	Renal artery stenosis
**COX**	Cytochrome c oxidase subunit
**ETC**	Electron transport chain
**OXPHOS**	Oxidative phosphorylation
**FGF21**	Fibroblast growth factor 21
**hMSCs**	Human Mesenchymal stem cell
**OPA1**	Optic atrophy 1
**Drp1**	Dynamin-related protein
**GSH**	Glutathione
**IL-13**	Interleukin-13
**p-STAT6**	Phosphorylated signal transducer and activator of Transcription 6
**IDH2**	Isocitrate dehydrogenase [NADP (+)] 2
**mt-COX3**	Mitochondrially encoded cytochrome c oxidase subunit 3
**STCs**	Scattered tubular-like cells
**mtROS**	Mitochondrial-reactive oxygen species
**SOD2**	Superoxide dismutase 2
**TSCC**	Tongue squamous cell carcinoma
**MnSOD**	Manganese superoxide dismutase
**MFN1**	Mitofusin-1
**MFF**	Mitochondrial fission factor
